# A Meso-Scale Computational Framework for Predicting Fracture Mechanisms in 3D-Printed Bouligand Cementitious Metamaterials

**DOI:** 10.3390/ma19132892

**Published:** 2026-07-06

**Authors:** Xuelian Yuan, Yaqing Jiang, Huiting Xiong

**Affiliations:** School of Civil Engineering, Wanjiang University of Technology, Ma’anshan 243031, China

**Keywords:** 3D concrete printing, Bouligand architecture, finite element analysis, cohesive zone model, damage tolerance, topological toughening

## Abstract

**Highlights:**

- Filament-resolved meso-scale FE models explicitly capture 3D-printed crack kinemat-ics.
- Bio-inspired Bouligand architectures shift cementitious failure to pseudo-ductile.
- 15° topologies enhance energy absorption >13-fold with <12% strength reduction.
- Step-wise crack twisting mechanisms remain robust against 30% interfacial degrada-tion.

**Abstract:**

The inherent brittleness of cementitious materials presents a fundamental limitation for advanced structural applications. While bio-inspired Bouligand architectures have demonstrated remarkable damage tolerance in natural composites, their systematic translation to brittle inorganic binders via 3D concrete printing (3DCP)—and the development of high-fidelity meso-scale models to quantitatively map the resulting strength–toughness design space—remains underexplored. This study aims to decouple the intrinsic topological toughening potential of helicoidal Bouligand architectures from the stochastic defects inherent to additive manufacturing, through a meso-scale finite element (FE) framework. To physically validate the model, a nano-clay-assisted rheological strategy was utilized to enable the support-free fabrication of these helicoidal prototypes. Computationally, a meso-scale FE framework integrating the concrete damaged plasticity (CDP) model with three-dimensional cohesive zone elements was developed to explicitly resolve inter- and intra-layer interfacial crack kinematics. Coupled physical compression tests and numerical simulations indicate that the 15° Bouligand architecture achieves a computationally predicted 16.3-fold increase in volumetric energy absorption (experimentally: 13.7-fold) compared to the 0° unidirectional baseline, with a modest ~11% reduction in compressive strength (from ~33.0 MPa to ~29.5 MPa in simulations; ~12% experimentally). Furthermore, numerical parametric studies across the complete pitch-angle design space reveal an optimal topological window at 15–30°, wherein the competing effects of crack deflection and structural integrity are balanced. Imperfection sensitivity analysis demonstrates that the topological toughening mechanism is relatively robust: even with a 30% reduction in inter-filament bonding strength, the work of fracture remains 12.4 times higher than that of the 0° control. These findings suggest that spatial toolpath programming offers a viable, geometry-driven strategy for developing damage-tolerant cementitious composites, complementing conventional material-level reinforcement approaches.

## 1. Introduction

### 1.1. The Intrinsic Dilemma of Inorganic Binders

Inorganic non-metallic cementitious materials (e.g., ordinary Portland cement, geopolymers, and ceramics) constitute the most consumed synthetic materials globally, forming the backbone of modern infrastructure [1]. However, constrained by their rigid ionic and covalent bond networks, these materials are inherently prone to brittle failure under tensile or impact loading. For over a century, structural engineering has relied on macroscopic reinforcement strategies, such as the incorporation of steel rebars or fibers, to arrest crack propagation. Nevertheless, this conventional toughening approach is often limited by weak interfacial bonding and non-uniform fiber dispersion [2,3]. Mitigating this intrinsic brittleness without significantly altering the primary chemical composition remains a persistent challenge in material engineering.

### 1.2. Bio-Inspired Topological Design

Confronted with this dilemma, natural biological structures offer effective “bottom-up” structural templates. Biological armors, such as molluscan nacre and the stomatopod dactyl club, are primarily composed of fragile inorganic minerals [4,5]. Yet, despite their brittle constituents, these structures exhibit remarkable damage tolerance derived from their hierarchical spatial architectures (e.g., Bouligand twisted plywood structures). This suggests that geometric and topological design can decouple the macroscopic mechanical performance from the intrinsic limitations of the base material. Translating these bio-inspired architectures into cementitious materials offers a viable pathway to develop damage-tolerant structural metamaterials [6,7].

In this context, we define “bio-inspired metamaterials” as architected materials whose meso-scale topology mimics biological structural designs (e.g., Bouligand twisted plywood) to achieve mechanical properties that exceed those predicted by mixture rules for their constituent phases [6,7].

#### 1.2.1. Prior Work on Architected Cementitious Materials

Several recent studies have explored the intersection of bio-inspired design and additive manufacturing of cementitious materials. Moini et al. [6] demonstrated that Bouligand-inspired architectures printed via extrusion could enhance the work of fracture of cement pastes, primarily through crack deflection at the meso-scale. Fan et al. [7] recently reviewed the current status of bio-inspired toughening strategies in 3D-printed cement, identifying pitch-angle optimization and interfacial engineering as the two dominant levers. On the computational side, Suksangpanya et al. [8] developed analytical and FE models of crack twisting in Bouligand architectures, though these were validated for polymer-matrix composites rather than brittle cementitious systems. Yang et al. [9] recently proposed a saddle-stitching strategy for interfacial toughening in 3D-printed concrete. However, significant gaps remain: (a) Systematic pitch-angle mapping—prior experimental studies tested only one or two pitch angles; a systematic computational sweep of the complete design space with explicit interfacial resolution has not been performed for cementitious systems. (b) Meso-scale interfacial resolution—existing numerical models of 3D-printed concrete predominantly employ homogenized continuum approaches [2,10,11] that obscure toolpath-induced anisotropy and the discrete nature of interfacial crack deflection. (c) Defect sensitivity—the robustness of topological toughening to the inherent interfacial defects of layer-by-layer extrusion (cold joints, variable bond strength) has not been quantitatively evaluated. A comparative summary of prior studies is provided in Table 1.

#### 1.2.2. Limitations of Existing Modeling Approaches

Current computational approaches to 3D-printed concrete fracture can be broadly classified into: (i) homogenized continuum damage models, which treat the printed material as an equivalent orthotropic medium and cannot resolve individual filament-level crack paths [2,10]; (ii) macro-scale cohesive zone models that assume predefined crack planes and are therefore incapable of capturing the stochastic crack deflection inherent to Bouligand architectures [12,13]; and (iii) detailed meso-scale models that explicitly discretize filaments and interfaces [8], which—while computationally expensive—are necessary to resolve the topological toughening mechanisms of interest. The present study adopts the third approach and, to the authors’ knowledge, represents the first application of an explicitly filament-resolved 3D cohesive network to a systematic parametric sweep of Bouligand pitch angles in a cementitious system.

### 1.3. Rheological Challenges in Additive Manufacturing

Translating these complex micro/meso-scale bio-inspired topologies into macro-scale cementitious monoliths is difficult to achieve using traditional formwork casting. Extrusion-based 3D concrete printing (3DCP) has emerged as an effective manufacturing approach to realize such spatial programming [15]. Yet, this introduces a critical rheological challenge. On the one hand, to ensure continuous extrusion through the nozzle, the ink must exhibit low apparent viscosity under high shear rates (shear-thinning behavior). On the other hand, immediately upon deposition, the material must undergo rapid structural reconstruction to develop a sufficiently high yield stress to resist gravity-induced deformation [16,17]. Balancing these transient rheological states remains a primary technical hurdle in printing complex, free-standing networks.

In the broader context of 3DCP, extrusion-based methods offer geometric freedom but introduce pronounced mechanical anisotropy due to the layered fabrication process. Inter-layer interfaces—often referred to as “cold joints”—typically exhibit 15–35% lower strength than the bulk filament material [14,18,19]. This anisotropy, while generally considered a liability, can potentially be harnessed as a toughening mechanism if the spatial arrangement of these weak interfaces is deliberately programmed—a key premise of the present work.

### 1.4. Our Strategy and Contributions

In this study, we utilize nano-clay-assisted thixotropic modulation—a well-established strategy for printable cementitious materials [18,20,21]—to address the competing rheological demands and enable the fabrication of helicoidal filament architectures with sufficient shape fidelity for the meso-scale geometric assumptions employed in the computational models.

This work is strictly positioned as a computation-driven mechanistic investigation. Because high-resolution, in situ physical imaging (such as micro-CT or 3D Digital Image Correlation) of complex internal crack twisting within dense, brittle cementitious matrices remains a significant experimental challenge, this computational framework acts as a critical “virtual laboratory.” Physical uniaxial compression tests are conducted purely to calibrate the macroscopic load–displacement baseline; once anchored by these metrics, the validated numerical model explores the unobservable micro-mechanisms.

The novel contributions are as follows: (1) Meso-scale FE framework with explicit interfacial resolution: We develop and validate a 3D finite element model that explicitly discretizes individual cementitious filaments and their inter-/intra-layer cohesive interfaces (CZM), enabling direct numerical observation of toolpath-level crack deflection kinematics. (2) Systematic mapping of the pitch-angle design space: We conduct the first parametric sweep of Bouligand architectures in a cementitious system with fully resolved interfacial mechanics, identifying an optimal topological window (*γ* = 15–30°). (3) Quantitative imperfection sensitivity analysis: We numerically demonstrate that the topological toughening mechanism is robust to interfacial bond degradation (up to 30%), with mechanistic explanation via compensatory crack tortuosity increase.

The following established techniques are employed but are NOT claimed as novel: nano-clay thixotropic modification [17,20], the CDP constitutive model [22,23], the CZM framework [12,13], the B-K mixed-mode criterion [24], and the Bouligand architectural concept [4,6,8].

### 1.5. Research Objectives and Hypotheses

Based on the identified research gaps (Section 1.2.1 and Section 1.2.2), this study addresses the following research questions:

(1) RQ1: Can helicoidal Bouligand architectures with small pitch angles (*γ* = 15–30°) shift the compressive failure mode of 3D-printed cementitious materials from brittle longitudinal splitting to pseudo-ductile progressive damage?
(2) RQ2: What is the quantitative relationship between pitch angle and the strength–toughness trade-off, and does an optimal topological window exist?
(3) RQ3: To what extent is the topological toughening mechanism sensitive to the interfacial bond degradation inherent to layer-by-layer extrusion?

The corresponding testable hypotheses are:

(1) H1: The 15° Bouligand architecture produces a >10-fold enhancement in volumetric energy absorption compared to the 0° control, at the cost of a modest (<15%) reduction in compressive strength.
(2) H2: The maximum toughening effect occurs at intermediate pitch angles (*γ* = 15–30°), where the orientational mismatch is sufficient to deflect cracks without severely compromising structural integrity.
(3) H3: The topological toughening mechanism is relatively insensitive to moderate (≤30°) reductions in interfacial bonding strength, owing to compensatory increases in crack path tortuosity.

The systematic study is organized as follows: Section 2 details the material formulation and experimental testing setups. Section 3 introduces the computational modeling framework. Section 4 presents the micro-mechanisms of ink rheology, the numerical and experimental fracture results, and a parametric analysis to map the topological design space. Section 5 provides a comprehensive discussion on the topological toughening mechanisms, imperfection sensitivity, alternative failure modes, and model limitations. Finally, Section 6 draws the conclusions.

## 2. Materials and Experimental Methods

### 2.1. Raw Materials and Mixture Preparation

The cementitious ink was prepared using Ordinary Portland cement (CEM I 52.5R) as the primary binder. The cement was supplied by Anhui Conch Cement Co., Ltd. (Wuhu, China). It has a Blaine fineness of 410 m^2^/kg. The chemical composition of the cement is listed in Table 2. The NM was supplied by Zhejiang Fenghong New Material Co., Ltd., Huzhou China (Product Grade: Highly Purified Sodium Montmorillonite), with a purity of 95% montmorillonite. Its essential characteristics include a cation exchange capacity (CEC) of 98 meq/100 g and a BET specific surface area of 45 m^2^/g. The particle size distribution (PSD) of the dry powder is characterized by *d*_10_ = 2.5 μm, *d*_50_ = 12.0 μm, and *d*_90_ = 35.0 μm. A polycarboxylate ether-based superplasticizer (PCE) was used for initial particle dispersion. The PCE was supplied by Jiangsu Sobute New Materials Co., Ltd., Nanjing China (product code: SBT-PCA(I)), with a solid content of 40 wt%. To compensate for the high water demand of the nano-clay and maintain extrudability at a water-to-binder ratio of 0.32, the PCE dosage was set at 0.20% by weight of the binder (calculated as active solid content).

The water-to-binder (w/b) ratio was 0.32, and the NM dosage was 0.8% by weight of the binder. The mixing protocol was strictly controlled to ensure a consistent initial rheological state: (i) NM was pre-dispersed in deionized water and ultrasonicated (240 W, 40 kHz) for 15 min; (ii) the colloidal suspension was combined with the PCE solution; (iii) cement powder was gradually added while mixing in a standard planetary mixer (Model JJ-5, Wuxi Jianyi Instrument & Machinery Co., Ltd., Wuxi, China) at a low speed of 140 RPM for 2 min; (iv) the mixing speed was then increased to a high speed of 285 RPM for 2 min; (v) the paste was allowed to rest for 1 min to facilitate PCE adsorption; (vi) a final high-shear mixing at 285 RPM for 1 min was executed immediately before printing or rheological measurement to eliminate any false set and establish a uniform dynamic reference state.

### 2.2. Rheological Characterization

Rheological measurements were conducted using a rotational rheometer (Model MCR 302, Anton Paar GmbH, Graz, Austria) at 20 ± 1 °C. A 4-blade vane rotor combined with a serrated cylindrical cup was utilized to mitigate wall-slip effects.

For steady-state flow curves, samples were pre-sheared at 100 s^−1^ for 60 s to eliminate shear history, followed by a logarithmic shear rate sweep from 0.01 to 200 s^−1^. The dynamic structural recovery kinetics were evaluated via a three-interval thixotropy test (3ITT) [16,20], consisting of: (1) a rest stage at 0.01% oscillatory strain for 60 s; (2) a high-shear breakdown stage at 100 s^−1^ for 30 s; and (3) a recovery stage returning to the 0.01% strain for 60 s, recording the storage (*G*′) and loss (*G*″) moduli.

### 2.3. Zeta Potential Measurement and DLVO Theoretical Modeling

To elucidate the micro-mechanisms of the nanoclay-assisted thixotropy, the interparticle interaction energy profiles were calculated based on the classic Derjaguin–Landau–Verwey–Overbeek (DLVO) theory. The total interaction energy (*V*_total_) was determined as the sum of the van der Waals attraction (*V*_vdW_) and the electrostatic double-layer repulsion (*V*_edl_).

For the theoretical calculation, the effective Hamaker constant (*A*) for the cement–nanoclay–water interacting system was assigned a value of 1.5 × 10^−20^ J, consistent with typical silicate-based colloidal suspensions [25,26]. To quantify the electrostatic repulsive forces, the zeta potential (*ζ*) of the formulated suspension was measured using electrophoretic light scattering (Model Zetasizer Nano ZS, Malvern Panalytical Ltd., Malvern, UK) at 20 °C. To prevent the artificial dissolution of ions and maintain the native chemical environment during measurement, the samples were diluted using a synthetic cement pore solution rather than deionized water [27]. This synthetic pore solution was formulated to replicate the typical early-age ionic environment of the OPC mixture, consisting of 0.35 mol/L KOH, 0.10 mol/L NaOH, and saturated with Ca(OH)_2_ and CaSO_4_ [28]. Under this specific high-ionic-strength environment, the average zeta potential (*ζ*) of the formulated colloidal suspension was measured to be −14.5 ± 1.2 mV.

Given the high ionic strength of this pore solution—dominated by Ca^2+^, K^+^, Na^+^, and OH^−^ ions—the electrical double layer is highly compressed. Accordingly, the Debye screening length (*κ*^−1^) was calculated to be approximately 1.2 nm based on the ionic concentration. These experimentally derived and literature-validated parameters [25,26] were subsequently substituted into the DLVO governing equations to generate the interaction energy versus distance curves discussed in Section 4.1.

### 2.4. Validation of Printability and Shape Retention

Specimens were printed using a custom-built 3-axis gantry 3D printer equipped with a progressive cavity pump and a 5.0 mm circular nozzle. The volumetric extrusion rate was 35 mL/min, synchronized with a constant nozzle travel speed of 30 mm/s. The as-printed filament width was measured as 6.5 ± 0.3 mm (mean ± SD, *n* = 10). The layer height was maintained at 2.5 mm. The interlayer time interval was 15 s per layer, and the total number of layers per slab was 40. All printing was conducted under ambient laboratory conditions (*T* = 20 ± 2 °C, RH = 55 ± 5%).

To ensure geometric consistency with the idealized FE models and to avoid boundary defects associated with frequent nozzle starts/stops in small circular paths [13], large rectangular slab specimens were first printed via G-code utilizing straight parallel toolpaths with predefined pitch angles (*γ*) of 0°, 15°, and 30°. After 28 days of standard curing (>95% RH, 20 ± 2 °C), cylindrical samples (50 mm diameter, 100 mm height) were extracted from the central region of the printed slabs using a wet core drilling machine (Model HZ-15, Wuxi Jianyi Instrument & Machinery Co., Ltd., Wuxi, China). To verify shape fidelity, cross-sectional images of as-printed filaments were captured using a digital microscope (Dino-Lite, AnMo Electronics Corporation, New Taipei City, Taiwan). The measured layer height (2.5 ± 0.2 mm) and filament width (6.5 ± 0.3 mm) confirmed geometric deviations of <8% from target values, providing the experimental basis for the idealized geometric assumptions in the computational models.

This physical printing campaign served as a targeted validation of the ink’s rheological capacity to resolve complex helicoidal toolpaths with high shape fidelity. By successfully fabricating free-standing specimens with a 100 mm height and complex internal features without perceptible gravitational deformation, the effectiveness of the nano-clay-assisted thixotropic modulation was demonstrated. We emphasize that “free-standing” here refers specifically to the absence of temporary supports during printing, enabled by the rapid thixotropic build-up [11]; it does not imply geometric perfection.

This methodological separation—verifying printability experimentally while evaluating fracture mechanics numerically—constitutes a deliberate strategy to isolate the influence of spatial topology on damage tolerance. By confirming that the material’s structural build-up can faithfully translate digital designs into structural domains, these trials provide a physical basis for the idealized geometric assumptions employed in the subsequent meso-scale finite element framework. This approach ensures that the identified toughening mechanisms are attributable to the programmed topology rather than being obscured by manufacturing-induced stochastic defects [13].

### 2.5. Mechanical Testing

Cylindrical specimens (0° and 15°) were subjected to uniaxial compression (ASTM C39/C39M–21) after 28 days of standard curing. Uniaxial compression was selected as the primary loading mode because: (i) it represents the dominant service loading for structural concrete elements, and (ii) the transverse tensile strains induced by the Poisson effect (*ν* ≈ 0.2) provide a well-defined driving force for interfacial crack initiation and longitudinal splitting along the cold joints [10,29], which is mechanistically analogous to the mode-I opening that governs Bouligand toughening in tension [8]. However, we acknowledge that flexural and fracture toughness tests are more sensitive probes of crack-deflection mechanisms, which will be essential in future investigations.

Testing was conducted using a 300 kN universal testing machine (Model CMT5305, MTS Systems (China) Co., Ltd., Shenzhen, China) under displacement control at a constant rate of 0.5 mm/min. Axial strain was precisely determined via two external linear potentiometers mounted symmetrically on opposite sides over a 50 mm gauge length. The average of the two external readings was reported (sampling rate: 10 Hz), while a built-in LVDT was utilized to monitor the gross crosshead displacement.

Regarding statistical considerations, a sample size of *n* = 3 per configuration was adopted, consistent with other exploratory 3DCP studies [13,18]. While this limits statistical power and the results should be interpreted with caution, all experimental quantities are rigorously reported as the mean ± standard deviation (SD) along with the coefficient of variation (CoV).

To provide a structured overview of the entire experimental campaign, the specific standards, specimen dimensions, and loading protocols for all fluid-state and hardened-state characterizations are comprehensively summarized in Table 3.

## 3. Computational Methodology

To simulate the fracture behavior of the 3D-printed metamaterials, a meso-scale finite element (FE) framework was developed in Abaqus/Explicit. Unlike macroscopic continuum models that assume homogenization, this meso-scale approach explicitly discretizes the structural geometry into bulk cementitious filaments and inter-/intra-layer interfaces to represent the toolpath-induced heterogeneity [34].

### 3.1. Constitutive Modeling of the Cementitious Matrix

**Figure 1:**
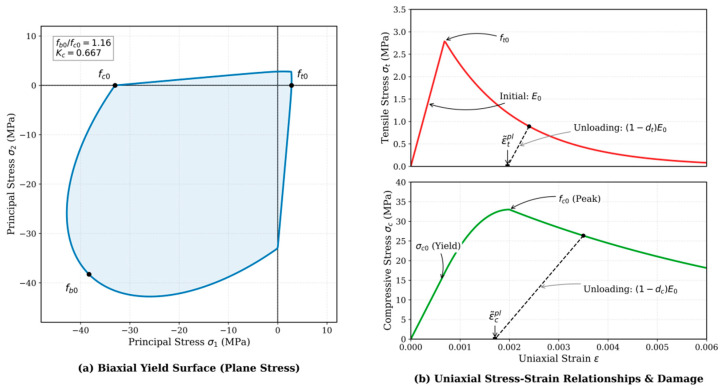
Schematic representation of the CDP model used for the bulk matrix: (**a**) the biaxial yield/failure surface in the plane stress state governed by *f*_b0_/*f*_c0_ and *K*_c_; (**b**) the theoretical stress–strain relationships and stiffness degradation under uniaxial tension and compression, characterized by damage variables *d*_t_ and *d*_c_.

To accurately capture the non-linear fracture mechanics of the 3D-printed bulk cementitious filaments, the concrete damaged plasticity (CDP) model was employed [22,23]. This continuum, plasticity-based damage model assumes that the main two failure mechanisms of cementitious materials are tensile cracking and compressive crushing. To provide a clear theoretical foundation for the utilized parameters, Figure 1 illustrates the failure surface and the uniaxial stress–strain relationships governing the matrix.

The failure criterion of the CDP model is defined by a multi-axial yield surface, which relies on a modified Drucker–Prager strength hypothesis. As depicted in Figure 1a for a plane stress state, the shape of the yield surface is primarily governed by two core plasticity parameters: the ratio of initial biaxial to uniaxial compressive yield stress (*f*_b0_/*f*_c0_), which defines the expansion of the failure envelope in the biaxial compression zone, and the parameter *K*_c_, which determines the shape of the failure surface on the deviatoric plane. Furthermore, the plastic flow is governed by a non-associated flow rule, utilizing the dilation angle (*ψ*) and flow potential eccentricity (*e*) to characterize the volumetric expansion under high shear stress.

Beyond the elastic limit, the progressive degradation of the material stiffness is modeled via isotropic damage mechanics. As shown in Figure 1b, the stress–strain responses under uniaxial tension and compression are characterized by strain-softening behavior. The degradation of the initial elastic stiffness (*E*_0_) is quantified by two independent damage variables: tensile damage (*d*_t_) and compressive damage (*d*_c_), which range from 0 (undamaged) to 1 (complete loss of load-bearing capacity). The effective stress ($\bar{\sigma }$) is related to the nominal stress (*σ*) via the damage formulation: *σ* = (1 − *d*) $\bar{\sigma }$, where *d* represents the corresponding scalar damage variable.

The specific constitutive parameters utilized in this study—including *ψ*, *e*, *f*_b0_/*f*_c0_, *K*_c_, and the damage initiation criteria—were assigned based on standard theoretical defaults mathematically established for the CDP yielding surface [35], validated against typical values for high-performance 3D-printed cementitious composites [10], and finely tuned via our experimental calibrations, as summarized in Table 4.

### 3.2. Cohesive Zone Modeling of the 3D Printing Interfaces

Extrusion-based 3D printing introduces structural anisotropy due to the formation of inter-filament interfaces. These weak boundaries were explicitly modeled using the Cohesive Zone Model (CZM) governed by a bilinear traction-separation constitutive relationship [13]. The mechanical behavior of the cohesive elements assumes initial linear elastic behavior before damage, followed by a linear softening process upon reaching the damage threshold.

Damage initiation at the interfaces, representing the onset of micro-cracking, was determined by the quadratic nominal stress criterion. Given the multi-axial stress states present in the loaded 3D-printed architectures, damage initiates when the stress ratio sum reaches unity:

(1) $${\left\{\left.\frac{\left\langle \left.t_{n}\right\rangle \right.}{\tau_{n}}\right\}\right.}^{2}+{\left\{\;\left.\frac{t_{s}}{\tau_{s}}\;\right\}\right.}^{2}+{\left\{\;\left.\frac{t_{t}}{\tau_{t}}\;\right\}\right.}^{2}=1$$

where *t*_n_, *t*_s_, and *t*_t_ are the current nominal tractions in the normal and two local shear directions, respectively. The Macaulay bracket $\left\langle \left.\cdot \right\rangle \right.$ ensures that pure compressive stress does not initiate cohesive damage. The terms *τ*_n_, *τ*_s_, and *τ*_t_ (provided in Table 4) represent the corresponding normal peak strength and shear peak strengths of the interfaces, reflecting the specific cold-joint bonding properties induced by the extrusion time gap and surface roughness.

Post-initiation damage evolution and ultimate debonding were controlled by an energy-based fracture criterion. In the Bouligand architectures (15° and 30°), the layer-by-layer rotational misalignment forces cracks to deflect out-of-plane and twist, creating a highly mixed-mode fracture (combination of tension and in-plane/out-of-plane shear). To accurately account for this complex mechanism, the Benzeggagh–Kenane (B-K) damage evolution law was adopted [24]. The B-K criterion defines the equivalent critical fracture energy ($G_{equiv}^{C}$) under mixed-mode loading as:

(2) $$G_{equiv}^{C}=G_{IC}+(G_{IIC}-G_{IC}){\left(\frac{G_{shear}}{G_{total}}\right)}^{n}$$

where *G_I_* is the work done by the normal traction, and *G*_shear_ = *G*_II_ + *G*_III_ is the work done by the shear tractions, summing to the total dissipated energy *G*_total_. As detailed in Table 4, *G*_IC_ is the Mode I (opening) fracture energy, while *G*_IIC_ and *G*_IIIC_ are the Mode II/III (sliding/tearing) fracture energies. The parameter *η* is a cohesive property material constant that dictates the sensitivity of the failure envelope to the mode mixity. While originally developed for polymer composites, the B-K criterion has been successfully extended to simulate mixed-mode debonding in 3D-printed cementitious interfaces [12,13]. Based on the quasi-brittle nature of the cementitious matrix and our inverse analytical calibrations, *η* was assigned a value of 1.6, ensuring that the model accurately captures the experimentally observed frictional slip and step-wise crack branching.

### 3.3. Spatial Discretization and Solver Configuration

**Figure 2:**
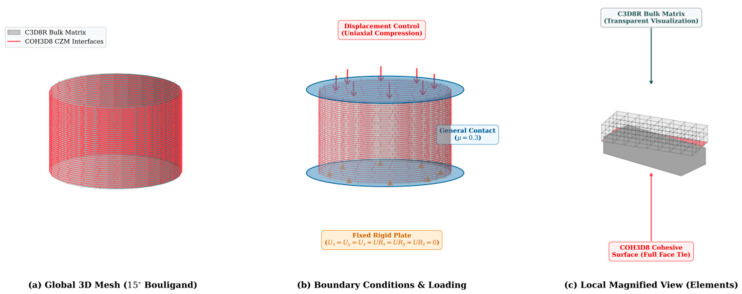
Finite element model assembly and boundary conditions for quasi-static compression (15° Bouligand architecture). (**a**) Global 3D mesh showing discretized cementitious filaments and embedded cohesive interfaces. (**b**) Boundary conditions featuring a fixed bottom rigid plate and a displacement-controlled top plate. The red arrows indicate the applied downward displacement, and the yellow triangles represent the fully fixed boundary conditions at the bottom. (**c**) Local magnified view detailing the C3D8R bulk elements and zero-thickness COH3D8 cohesive elements. In the visualizations, the grey color represents the cementitious bulk elements, while the red color indicates the cohesive interfaces.

To explicitly demonstrate the computational setup and boundary conditions, Figure 2 illustrates the full 3D finite element assembly using the representative 15° Bouligand model as an example. The bulk filaments were meshed using 8-node linear brick elements with reduced integration (C3D8R), and the interfaces were discretized with 8-node cohesive elements (COH3D8). As shown in Figure 2, the architected cylindrical specimen is positioned between two analytical rigid plates. The bottom plate was fully constrained in all translational and rotational degrees of freedom to act as a rigid base. Uniaxial compression was simulated by applying a downward displacement to the top rigid plate. Furthermore, to prevent unphysical interpenetration of the filament elements after the deletion of fully damaged cohesive interfaces, a general self-contact algorithm was implemented. This incorporated a “hard” contact formulation for normal behavior and a penalty friction formulation (with a friction coefficient of *μ* = 0.3) for degraded cementitious interfaces [18,35] for tangential sliding.

Due to the high degree of nonlinearity associated with progressive cracking, extensive interfacial delamination, and complex self-contact, the Abaqus/Explicit dynamic solver was utilized for the quasi-static simulation. The quasi-static condition was maintained by applying a smooth step amplitude to the displacement load and utilizing controlled mass scaling. The kinetic energy of the entire system was continuously monitored to ensure it remained below 5% of the total internal energy throughout the loading history, confirming that inertial effects were negligible [35].

### 3.4. Mesh Sensitivity Analysis

When simulating strain localization using continuum damage models like CDP, the macroscopic response often exhibits inherent mesh dependency [36]. To address this and determine the spatial resolution required for the discrete CZM, a mesh sensitivity analysis was performed on the 0° unidirectional control model.

**Figure 3:**
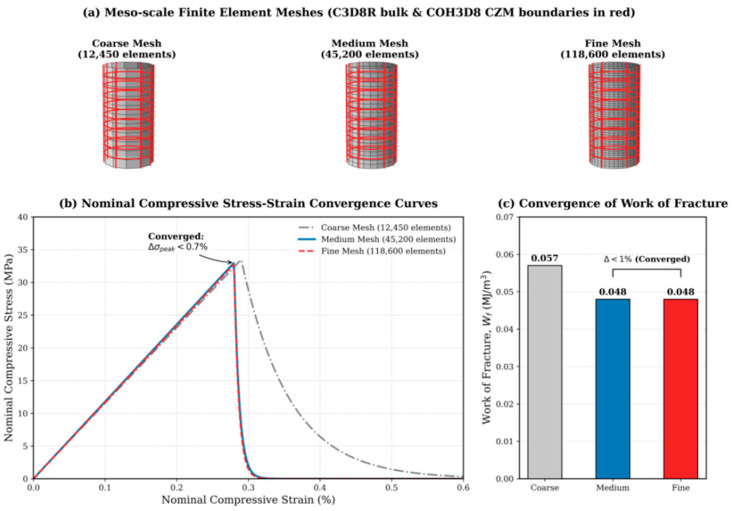
Mesh sensitivity analysis of the 0° unidirectional control model. (**a**) Spatial discretization of the finite element meshes. (**b**) Simulated nominal compressive stress–strain convergence responses. (**c**) Calculated volumetric work of fracture (*W*_f_). Numerical convergence is achieved at the medium mesh density.

Three discretization densities were compared and visually presented in Figure 3a: a coarse mesh (12,450 elements), a medium mesh (45,200 elements), and a fine mesh (118,600 elements). The corresponding nominal compressive stress–strain curves are plotted in Figure 3b.

The coarse mesh yields an artificially stiffened pre-peak response and overestimates the ultimate compressive strength (~33.5 MPa). This overestimation stems from insufficient element resolution within the fracture process zone (FPZ), which kinematically constrains damage evolution [37]. By refining the model to the medium mesh, the peak stress converges to approximately 33.0 MPa, and the post-peak brittle load drop is resolved more clearly.

Further refinement to the fine mesh results in a peak strength of ~32.8 MPa, representing a relative deviation of less than 0.7% from the medium mesh. The macroscopic strain-softening trajectories of the medium and fine meshes show close agreement, suggesting that numerical convergence is reasonably approximated, as further confirmed by the stabilized work of fracture depicted in Figure 3c.

It is worth noting that while mesh refinement traditionally alters strain localization and crack propagation paths in standard continuum damage mechanics, the macroscopic crack trajectories within our CZM-governed modeling framework are topologically predetermined by the explicitly discretized inter-layer weak interfaces [38]. Consequently, refining the bulk CDP elements enhances the localized stress gradient resolution within individual filaments but does not alter the global macroscopic failure pathways, rendering the simulated damage contours visually identical across the coarse, medium, and fine mesh densities. Therefore, numerical convergence in this specific discrete-interface framework is rigorously evaluated and verified through macroscopic stress–strain trajectories and quantitative energetic stabilization (*W*_f_), rather than visually redundant geometric profiles.

Given the substantial computational cost associated with explicitly evaluating the 3D cohesive interfaces, the medium mesh (~45,200 elements) provides an acceptable compromise between numerical accuracy and solving efficiency. Consequently, this discretization level was applied to all subsequent parametric models.

### 3.5. Quasi-Static Energy Balance Verification

The numerical simulation of extensive cohesive interfacial debonding, coupled with progressive matrix cracking, introduces severe material and geometric nonlinearities. Consequently, the explicit dynamic solver was utilized to circumvent the convergence difficulties inherent in implicit integration schemes. However, when employing an explicit dynamic algorithm for a quasi-static boundary value problem, it is imperative to verify that inertial forces do not artificially dominate the structural response.

To ensure quasi-static conditions, a smooth step amplitude curve was applied to the displacement boundary condition, thereby mitigating initial acceleration shocks. Furthermore, the global energy balance of the model was continuously monitored. The validity of the quasi-static assumption requires that the kinetic energy (ALLKE) remains a negligible fraction—typically below a 5% threshold—of the total internal energy (ALLIE) for the majority of the deformation process [35].

As detailed in Figure S1 of the Supplementary Information, the internal energy scales quadratically during the initial elastic phase and subsequently transitions to a nearly linear increase as widespread macroscopic damage occurs. Concurrently, the kinetic energy exhibits minor high-frequency oscillations characteristic of explicit solvers but remains consistently bounded near zero. Throughout the entire loading history, the kinetic energy strictly resides well below the 5% threshold of the internal energy (reaching a maximum of approximately 0.4%). These energetic metrics confirm that the simulated fracture mechanisms are driven by the quasi-static strain energy release, rather than artifacts of dynamic stress wave propagation or inertial effects.

### 3.6. Geometric Modeling of Bio-Inspired Architectures

As will be experimentally demonstrated by the rheological characterizations in Section 4.1, the formulated ink exhibits a rapid sol-to-gel transition and near-instantaneous shape retention. This thixotropic build-up physically prevents structural sagging during extrusion, providing the necessary empirical justification for employing the idealized, pristine meso-structures constructed in this section as our computational baseline.

**Figure 4:**
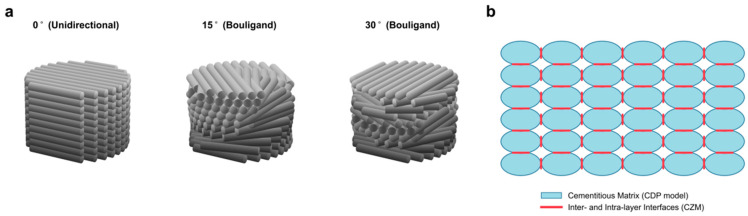
Geometric modeling and constitutive setup for the meso-scale finite element analysis. (**a**) 3D schematics of the 0° unidirectional control and the helicoidal Bouligand configurations (15° and 30° pitch angles). (**b**) Representative volume element (RVE) schematic illustrating the meso-scale spatial discretization, differentiating the bulk cementitious matrix (CDP model) and the explicit inter-/intra-layer interfaces (CZM).

Accordingly, 3D finite element (FE) models were developed to evaluate the progressive fracture behavior under quasi-static uniaxial compression. The Bouligand architecture was adopted as the primary topological prototype, defined by a constant pitch angle (*γ*) between successive printed layers [6]. Models with varying pitch angles (*γ* = 0°, 15°, 30°, 45°, 60°, and 90°) were generated to parallel the experimental program, with the 0° and helicoidal configurations conceptually illustrated in Figure 4a. To capture the multi-phase failure mechanisms detailed in Section 3.1, Section 3.2, Section 3.3, Section 3.4 and Section 3.5, the bulk matrix was assigned the Concrete Damaged Plasticity (CDP) model, while the inter-filament boundaries were explicitly resolved using a cohesive zone model (CZM) network (Figure 4b). This integrated geometric and constitutive configuration enables the multi-scale performance evaluations discussed sequentially in Section 4. It must be explicitly acknowledged early on that these geometric formulations assume perfect filament dimensions, precise spatial placement, and uniform interfacial properties. As physical 3DCP inherently introduces geometric defects (e.g., filament waviness, nozzle positioning errors, and local porosity) [39,40,41], the computational results derived from these idealized representative volume elements (RVEs) should be strictly interpreted as upper-bound estimates of the topological toughening potential.

## 4. Results and Discussion

### 4.1. Micro-Mechanisms of Ink Rheology and Thixotropic Programming

**Figure 5:**
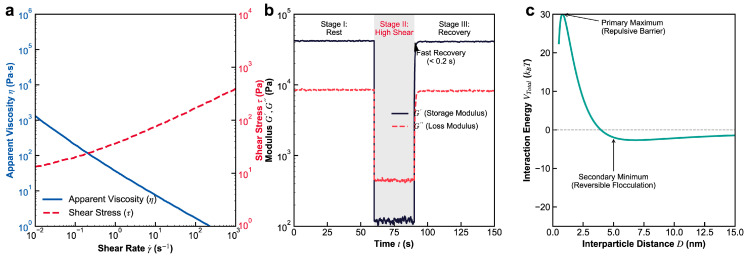
Rheological characterization and DLVO interaction energy of the 3DCP ink. (**a**) Steady-state flow curve showing apparent viscosity and shear stress versus shear rate. (**b**) Three-interval thixotropy test (3ITT) evaluating structural recovery via storage (*G*′) and loss (*G*″) moduli. (**c**) Calculated DLVO total interaction energy (*V*_total_) as a function of interparticle distance. Note: To maintain visual clarity of the continuous data streams, representative curves from triplicate independent measurements (*n* = 3) are presented.

The support-free additive manufacturing of the bio-inspired architectures relies on the spatiotemporal control of the ink’s viscoelasticity. As shown in the steady-state flow curves (Figure 5a), the formulated ink exhibits typical Bingham-pseudoplastic behavior. At near-equilibrium ($\dot{\gamma \;}\to 0$), the apparent viscosity exceeds 10^4^ Pa·s, providing initial shape retention. Under extrusion-induced shear, the viscosity decreases by over three orders of magnitude to <10^1^ Pa·s, allowing for smooth extrusion through the nozzle while maintaining a stable filament cross-section.

To correlate microscopic particle interactions with macroscopic printability, a three-interval thixotropy test (3ITT) was used to simulate the extrusion cycle (rest-shear-rest). As indicated in Figure 5b, the storage modulus (*G*′) demonstrates rapid structural recovery, regaining approximately 95% of its initial plateau value (*G*′_0_) within 0.2 s. This fast sol-to-gel transition ensures that the static yield stress is restored before the extruded filament undergoes significant gravitational deformation, thereby enabling structural self-support [18].

The physical origin of this rapid thixotropy was analyzed via DLVO theoretical modeling (Figure 5c), which aligns with the fundamental colloidal and micro-mechanical mechanisms reported in established literature [26]. Instead of relying on the relatively slow cement hydration process, the incorporation of highly anisotropic nano-clay particles alters the early-age interaction network. By modulating the surface zeta potential (*ζ*) to maintain optimal negative values, the electrostatic and van der Waals interactions between the cement grains and nano-clay platelets were adjusted. This hetero-coagulation system exhibits a secondary energy minimum (*V*_min_) at an interparticle distance of ~5 nm. Unlike irreversible primary-minimum aggregation, this secondary-minimum trapping promotes the formation of a reversible clay-cement percolation network [21,42]. Under high shear, mechanical energy overcomes this shallow potential well, causing network disruption. Upon shear cessation, the particles are recaptured into the secondary wells, leading to rapid scaffold reconstruction.

To ensure the reliability and quality of the raw rheological data, all measurements were conducted in triplicate (*n* = 3) under strictly controlled temperature conditions (20 ± 1 °C) following a consistent pre-shearing protocol. The coefficient of variation (CoV) for critical extracted parameters, such as the dynamic yield stress and structural recovery rate, was consistently within 5%. For visual clarity and to prevent obfuscation of the transient physical trends, representative continuous curves are plotted in Figure 5.

This micro-mechanistic re-flocculation defines the operational printability window. By matching these rheological parameters with the extrusion kinematics, the fluidity required for pumping is effectively separated from the static rigidity needed for shape retention. This rheological control provides the necessary material conditions for the layer-by-layer fabrication of the Bouligand architectures evaluated in the subsequent sections.

### 4.2. Damage Evolution and 3D Stress Field Distribution

To investigate the topological toughening mechanisms, the internal stress redistribution and damage evolution under quasi-static compression were analyzed. The progression of cracking was evaluated using the tensile damage variable (*d*_t_) of the CDP model and the degradation of the cohesive elements.

**Figure 6:**
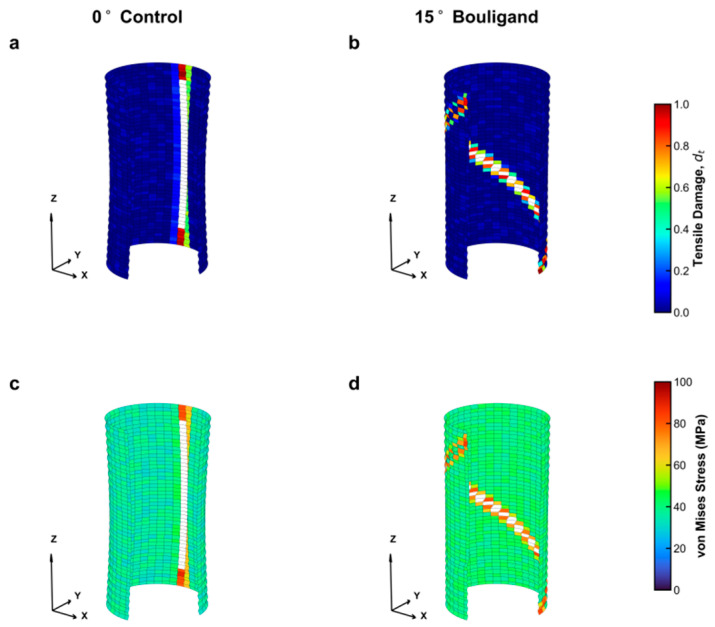
Meso-scale simulations of progressive failure under uniaxial compression. (**a**,**b**) Tensile damage (*d*_t_) contours of the 0° control and 15° Bouligand architectures. Elements with *d*_t_ > 0.95 are deleted to represent macro-cracks. (**c**,**d**) Corresponding von Mises stress distributions. All crack paths shown are computational predictions.

The computational results indicate that the failure modes depend strongly on the spatial topologies (Figure 6). In the unidirectional control group (0° architecture), compressive loading induces lateral tensile strains due to the Poisson effect, causing stress to localize along the continuous inter-filament interfaces. Once the local stress exceeds the cohesive strength, cracks initiate and propagate parallel to the printing direction. These continuous interfaces provide preferential paths for rapid crack growth, leading to longitudinal splitting and an abrupt loss of load-bearing capacity [10,29] (Figure 6a).

In the simulated Bouligand architectures (*γ* = 15° and 30°), the numerical framework predicts that crack propagation trajectories are actively deflected. Driven by the continuum mechanics formulations, initial micro-cracks are computed to nucleate at the intra-layer interfaces due to transverse tension. As these simulated crack fronts reach the adjacent, rotationally misaligned filament layer, their propagation is hindered. This orientation mismatch forces the cracks to deviate from their initial plane, inducing numerically predicted out-of-plane deflection and interfacial delamination.

Furthermore, the layer-by-layer rotation induces a 3D crack twisting mechanism, which represents the numerically predicted internal kinematics of the model rather than direct physical observations. The simulated macroscopic crack follows a staircase-like helical pathway guided by the shifting filament orientations, which significantly expands the theoretical fracture surface area [8]. Simultaneously, the model indicates that intact filaments positioned at divergent angles bridge the widening cracks, shielding the crack tips from critical stress concentrations (Figure 6b).

These predicted fracture behaviors correspond with the von Mises equivalent stress fields. Compared to the highly localized stress concentration in the 0° configuration (Figure 6c), the helicoidal architectures exhibit a dispersed stress distribution (Figure 6d). The applied load is transferred across the interwoven 3D filament network, mitigating localized strain peaks. Through these computationally derived mechanisms of successive crack deflection, bifurcation, and twisting, the Bouligand topology numerically shifts the failure mode from catastrophic brittle fracture to progressive damage, providing the theoretical basis for the continuous energy dissipation evaluated in the following section.

### 4.3. Experimental vs. Numerical Toughness Evaluation

**Figure 7:**
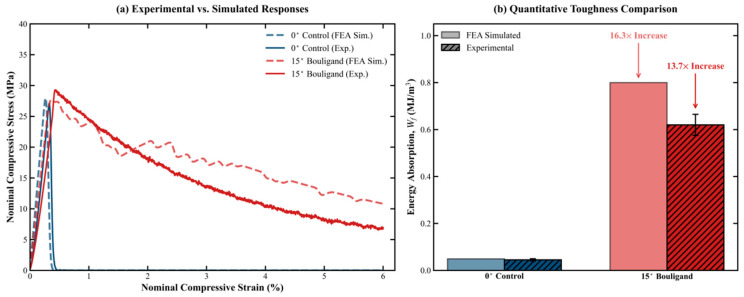
Macroscopic mechanical responses and quantitative toughness evaluation. (**a**) Nominal compressive stress–strain curves from physical experiments and FEA simulations. Experimental peak strengths are 30.2 ± 1.5 MPa (0° control) and 26.5 ± 2.1 MPa (15° Bouligand). (**b**) Volumetric energy absorption (work of fracture, *W*_f_) calculated up to a 6.0% strain limit. Error bars denote the standard deviation of experimental replicates (*n* = 3). The 13.7-fold toughness enhancement for the 15° configuration is statistically significant (*p* < 0.01).

To quantitatively evaluate the damage tolerance and validate the computational framework, Figure 7a compares the macroscopic stress–strain responses obtained from the FEA models and the physical uniaxial compression tests. Overall, the experimental observations agree with the numerical predictions regarding the transition in failure modes.

As shown in Figure 7a, both the experimental and simulated 0° control specimens exhibit typical brittle failure. After an initial non-linear compaction phase (toe region) observed in the physical specimens, the stress increases linearly up to the peak compressive strength (experimental ~30.2 MPa; simulated ~33.0 MPa), followed by a sharp load drop. This abrupt failure is associated with rapid longitudinal splitting, reflecting the intrinsic brittleness of the unidirectionally printed matrix.

In contrast, the 15° Bouligand architecture displays a pseudo-ductile softening behavior in both experimental and numerical results. While its initial stiffness is comparable to that of the control group, it retains a significant load-bearing capacity in the post-peak regime. The simulated curve shows discrete stress fluctuations corresponding to localized interfacial debonding and crack deflection. The experimental curve captures this energy-dissipating phase, displaying stick-slip features typical of progressive failure in interwoven architectures.

To quantitatively evaluate the damage tolerance, the volumetric energy absorption capacity (denoted herein as the volumetric work of fracture, *W*_f_) was calculated by integrating the nominal compressive stress–strain curves up to a predefined strain limit of 6.0% (Figure 7b). It must be explicitly noted that *W*_f_ in this context represents a macroscopic structural energy absorption metric, rather than an intrinsic material fracture toughness (e.g., Mode-I critical stress intensity factor, *K*_Ic_). The 6.0% integration limit was systematically selected based on three criteria: (i) it sufficiently exceeds the peak strain for all configurations, fully capturing the post-peak pseudo-ductile softening tail where the geometry-driven interfacial slip operates; (ii) it corresponds to a macroscopic structural displacement of approximately 6 mm, beyond which the 0° control specimens completely lose macroscopic integrity due to severe splitting; and (iii) it is consistent with the standard 5–10% integration bounds widely adopted in the recent literature for evaluating architected cementitious metamaterials [6,7]. This standardized cutoff ensures a fair and physically meaningful comparison across varying topologies by excluding the subsequent artificial densification phase of the heavily crushed debris. Based on this rigorously defined metric, the experimental peak strength (~26.5 MPa) and *W*_f_ (~0.62 MJ/m^3^) of the 15° specimens are lower than the simulated values (~29.5 MPa and 0.80 MJ/m^3^, respectively) (Table 5). This discrepancy is expected, as the numerical model assumes idealized filament geometry and perfect bonding, whereas physical 3D-printed specimens inevitably contain micro-pores and weaker cold joints.

Regarding the statistical dispersion inherent to the 3DCP process, the ultimate compressive strength exhibited a Coefficient of Variation (CoV) of 5.0% for the 0° control and 7.9% for the 15° architecture. For the volumetric work of fracture (*W*_f_), the CoV values were 17.8% and 6.8%, respectively. Although the sample size per configuration is relatively small (*n* = 3, consistent with exploratory 3DCP structural evaluations [13,14]), the magnitude of the topological toughening effect is substantial. An exploratory two-sample Welch’s t-test (assuming unequal variances) was conducted to compare the energy absorption capacities. The statistical analysis confirms that the 13.7-fold increase in experimental *W*_f_ for the 15° architecture is highly significant (*p* < 0.01). This rigorous statistical comparison indicates that the geometry-driven toughening mechanism effectively overcomes the inherent manufacturing variabilities.

Despite these differences, the topological toughening effect is evident in both approaches. The experimental results show a 13.7-fold increase in toughness for the 15° architecture compared to the 0° control (from 0.045 to 0.62 MJ/m^3^), which is in reasonable agreement with the 16.3-fold increase predicted numerically. However, because the computational framework omits real-world manufacturing anomalies (such as filament waviness, nozzle positioning errors, and local porosity) [39,40,41], this 16.3-fold numerical enhancement must be strictly treated as an upper-bound estimate rather than an expected practical performance level. Nonetheless, these findings confirm that spatial toolpath programming can effectively enhance the macroscopic toughness of 3D-printed cementitious materials.

### 4.4. Parametric Analysis on Orientational Mismatch—A Numerical Exploration

While Section 4.3 detailed the toughening mechanisms of the 15° Bouligand architecture, a broader parametric investigation is required to map the topological design space. An extended numerical sweep was conducted, varying the pitch angle (*γ*) from 0° to 90° (*γ* = 0°, 15°, 30°, 45°, 60°, and 90°). The objective is to evaluate the competing effects of orientational mismatch on macroscopic load-bearing capacity and energy dissipation.

**Figure 8:**
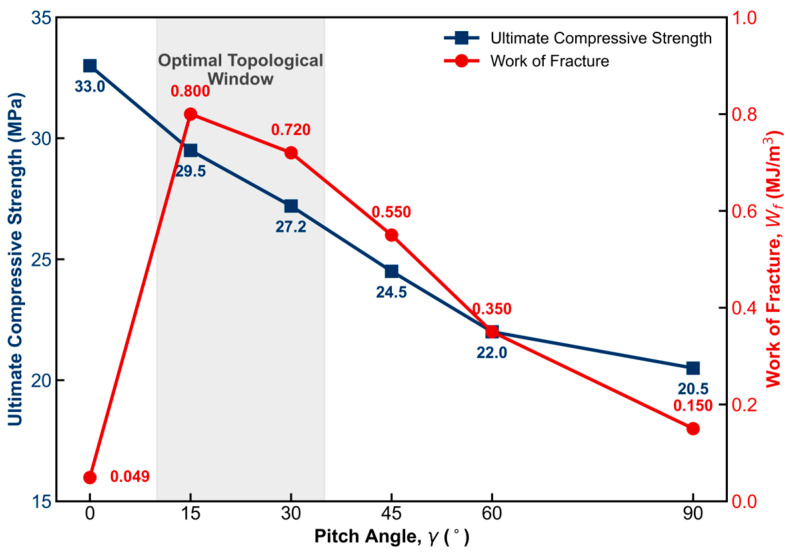
Variations in ultimate compressive strength and volumetric work of fracture (*W*_f_) across different pitch angles (*γ* = 0° to 90°). The gray shaded area indicates the optimal topological window (*γ* = 15° and 30°). The data presented in this figure represent numerical predictions only, without direct experimental verification.

The extracted ultimate compressive strength and volumetric work of fracture (*W*_f_) are plotted as functions of the pitch angle in Figure 8. The results indicate a structural trade-off governed by meso-scale anisotropy [34].

To provide a comprehensive overview of the strength–toughness trade-off induced by the topological programming, the macroscopic mechanical responses from both the physical calibration tests and the systematic numerical sweep are compiled in Table 6. As established in our methodology, physical testing (*n* = 3) was conducted for the 0° baseline and the 15° architecture to calibrate the energetic boundaries of the computational framework. The validated meso-scale FE model was subsequently utilized to predict the mechanical behavior across the remaining design space (30°, 45°, 60°, and 90°). As shown in Table 6, the numerical predictions for the 0° and 15° configurations exhibit excellent agreement with the experimental data (capturing the ~12% strength reduction and the >13-fold toughness enhancement), thereby demonstrating the fidelity of the computational framework in predicting the performance of the untested intermediate configurations.

As the pitch angle increases, the ultimate compressive strength decreases monotonically. The maximum strength is recorded in the 0° control (~33.0 MPa), where the applied principal stress aligns with the continuous vertical interfaces, maximizing the effective load-bearing area. Conversely, rotational misalignment (15° ≤ *γ* ≤ 90°) reduces the direct inter-layer contact area and introduces localized multi-axial stress states, particularly interlaminar shear [6]. This stress state promotes earlier cohesive debonding, leading to a gradual strength reduction down to ~20.5 MPa for the 90° cross-ply structure.

In contrast, the energy absorption capacity (*W*_f_) demonstrates a non-linear dependence on the pitch angle. Neither the 0° unidirectional nor the 90° cross-ply architecture yields maximum toughness. The 0° structure fails via rapid longitudinal splitting (*W*_f_ ≈ 0.049 MJ/m^3^). While the 90° structure arrests in-plane cracks, the orthogonal mismatch triggers extensive interfacial sliding before significant bulk deformation occurs, restricting its ultimate toughness (*W*_f_ ≈ 0.15 MJ/m^3^).

The maximum toughening effect emerges within a specific topological window of small pitch angles (*γ* = 15–30°) [8]. Within this range, the geometrical shift is sufficient to induce continuous, step-wise out-of-plane crack deflection and 3D twisting without prematurely compromising the global structural integrity. Specifically, the 15° architecture yields a high work of fracture (~0.80 MJ/m^3^) while retaining a compressive strength of ~29.5 MPa. These parametric findings provide quantitative guidelines for programming the spatial toolpaths of damage-tolerant cementitious composites.

## 5. Discussion: Topological Toughening Mechanisms and Meso-Scale Anisotropy

### 5.1. Brittle-to-Pseudo-Ductile Transition

The simulated stress–strain curves (Figure 7a) indicate a transition in failure mode governed by the print topology. For the 0° unidirectional control, the computational model predicts a sharp load drop after reaching the peak stress (~33.0 MPa). This discontinuity is associated with rapid longitudinal splitting along the aligned inter-filament interfaces, reflecting the brittle nature of the cementitious matrix.

In contrast, the 15° Bouligand architecture exhibits a pseudo-ductile response. The post-peak regime is characterized by a stochastic, step-wise softening behavior. These discrete load fluctuations correspond to localized cohesive debonding, inter-filament slip, and mechanical interlocking within the interwoven network. While peak strength is marginally reduced, the engineered interfaces facilitate frictional energy dissipation, yielding a predicted 16.3-fold increase in the volumetric work of fracture (*W*_f_).

This brittle-to-pseudo-ductile transition is mechanistically analogous to that observed in nacre-like alumina ceramics [43], where weak inorganic interfaces deflect cracks and generate extrinsic toughening. A key distinction in the present cementitious system is that the “weak” interfaces arise naturally from the layered extrusion process (cold joints) rather than requiring deliberate interphase engineering—suggesting that 3DCP’s inherent anisotropy, conventionally viewed as a defect, can be repurposed as a toughening asset through spatial toolpath design.

### 5.2. Crack Deflection and Trajectory Tortuosity

Homogenized continuum models generally predict macroscopic shear bands under compression. However, the explicitly modeled discrete interfaces in this study capture the tortuous fracture paths inherent to the 3D-printed meso-structure (Figure 6). In the helicoidal designs, the layer-by-layer rotation restricts in-plane crack propagation.

When transverse tensile cracks (induced by the Poisson effect) reach an inter-layer boundary, the pitch misalignment promotes out-of-plane crack deflection, branching, and arrest. As a result, the driving strain energy is distributed over a larger structural volume to create new fracture surfaces along the step-like Bouligand pathways, rather than driving a single localized splitting crack. This spatial redirection of damage mitigates sudden structural collapse, aligning with recent advanced topological strategies for interfacial toughening [7,9].

The crack deflection behavior can be evaluated using the He-Hutchinson criterion [44] and its extension by Parmigiani and Thouless [45]. While orthogonal crack impingement generally requires the interfacial toughness to be less than 25% of the bulk toughness to activate deflection [44], cracks impinging at oblique angles (*θ*) are more likely to deflect along the interface. In recent years, these classical interfacial fracture principles have been successfully leveraged to explain the damage tolerance and crack kinematics in 3D-printed architected cementitious and geopolymer materials [6,41,46]. In the 3D-printed Bouligand architectures, this impingement angle θ is determined by the pitch angle γ. Although the printed interfaces and the bulk matrix share comparable tensile strengths, the extrusion process inherently reduces the interfacial fracture energy (GIC). This reduction allows the cold joints to satisfy the deflection criteria under mixed-mode loading.

This relationship explains the observed peak in energy absorption at intermediate pitch angles (γ = 15–30°). At γ < 15°, interfacial delamination still occurs, but the small orientational mismatch restricts crack tortuosity, leading to limited energy dissipation. On the other hand, at γ > 30°, the larger misorientation induces pronounced out-of-plane crack deflection, but the associated reduction in inter-filament contact area prematurely compromises the overall load-bearing capacity.

### 5.3. Imperfection Sensitivity of the 15° Bouligand Architecture

**Figure 9:**
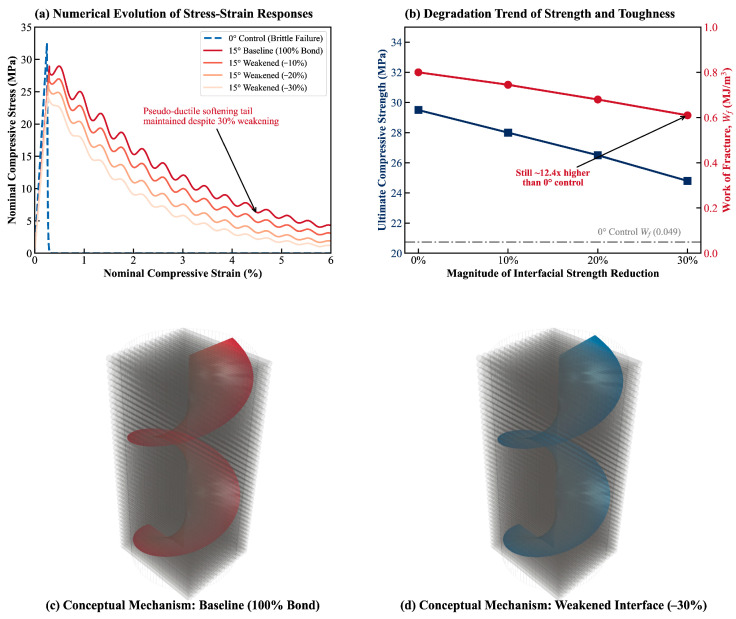
Imperfection sensitivity analysis of the 15° Bouligand architecture. (**a**) Simulated nominal compressive stress–strain responses under 0% to 30% interfacial strength reduction. (**b**) Degradation trends of ultimate compressive strength and volumetric work of fracture (*W*_f_). (**c**,**d**) Simulated 3D crack kinematics for the baseline (100% bond) and weakened (−30%) interfaces, respectively.The red color in (**c**) represents the continuous helical crack trajectory in the fully bonded baseline, whereas the blue color in (**d**) highlights the step-wise interfacial delamination path in the weakened model. (Note: Defects are represented herein solely through uniform interfacial strength reduction. Real 3DCP geometric anomalies are discussed in Section 5.5).

The cohesive properties assigned to the FEA models (Table 4) represent a baseline assumption for adequately bonded filaments. However, there is an intrinsic physical conflict governed by the 3DCP rheology. While the rapid thixotropic recovery (demonstrated via the 3ITT in Section 4.1) is crucial for shape stability, it concurrently induces a rapid escalation in the static yield stress of the substrate layer prior to the deposition of the subsequent layer. This swift microstructural stiffening inherently limits inter-filament polymer entanglement and binder hydration bridging. Consequently, weak inter-layer interfaces (cold joints) often occur due to the layer-by-layer extrusion process and varying time gaps [13,18]. To evaluate how these inherent defects affect the toughening mechanism of the 15° Bouligand design, a numerical parametric study was conducted. The nominal interfacial bonding strength was reduced by up to 30%, with the corresponding macro-mechanical responses and meso-scale crack kinematics presented in Figure 9. The degradation trends of ultimate compressive strength and volumetric work of fracture (*W*_f_) are summarized in Table 7.

The 30% lower bound for this parametric study is justified by two primary factors: (i) Rheological: The rapid thixotropic recovery demonstrated in Section 4.1 inherently limits the degree of cement hydration bridging across layers. Reported interlayer strength reductions in the 3DCP literature range from 15% to 35% [13,18,19]; and the high sensitivity of macroscopic fracture to such localized cohesive laws and print parameters has been extensively highlighted in recent computational frameworks [47]; therefore, 30% represents a realistic upper bound for typical printing conditions. (ii) Material: Beyond approximately a 30% reduction, the cohesive interface effectively degenerates to a friction-only contact, at which point the continuum-based assumptions of the CZM framework become less applicable.

The simulated stress–strain curves (Figure 9a) show that while the peak compressive strength decreases with the degradation of interfacial bonding, the pseudo-ductile softening tail is preserved. Unlike the 0° control group, which fails in a brittle manner shortly after the elastic limit, the 15° architecture maintains load-bearing capacity in the post-peak stage. Quantitatively (Figure 9b), even with a 30% reduction in interfacial strength, the retained *W*_f_ is approximately 12.4 times higher than that of the 0° benchmark. This suggests that the macroscopic toughening effect is relatively insensitive to local variations in interfacial bonding.

The origin of this defect tolerance can be explained through the idealized computational representative volume elements (RVEs) in Figure 9c,d. In the baseline model (100% bond, Figure 9c), the crack path follows a continuous helicoidal twist. However, when the interface is weakened (Figure 9d), the crack propagation mode changes. The lower bonding strength promotes interfacial slip, forming a horizontal delamination step before the crack deflects into the next layer at a mixed-mode kink angle of approximately 60°.

This step-wise crack propagation increases the overall crack tortuosity. The energy dissipated by the delamination process compensates for the loss of intrinsic interfacial bonding energy. These numerical results indicate that the 15° Bouligand architecture theoretically allows the material to maintain high energy dissipation capacity under uniform interfacial degradation.

### 5.4. Alternative Failure Mechanisms and Loading Conditions

The numerical results presented in Section 5.3 focus on the interface-governed crack deflection mechanism under unconfined compression. However, we acknowledge that alternative failure modes could become operative under different conditions. First, if interfacial bonding is exceptionally strong, cracks may propagate through bulk filaments rather than deflecting along interfaces, providing limited topological toughening benefit [44,45]. Second, at very small pitch angles (e.g., *γ* < 5°), the interlayer misorientation may be insufficient to deflect cracks, causing failure to transition from tensile splitting to localized compressive crushing. Finally, the current CDP parameters were calibrated for unconfined compression. Under laterally confined conditions in large-scale printed structures, damage evolution could differ significantly. Furthermore, while uniaxial compression evaluates the dominant service loading, flexural and mode-I fracture toughness tests are generally more sensitive probes for crack-deflection mechanisms [6,8]. Incorporating these varied loading scenarios represents a critical boundary for defining the true topological toughening regime.

### 5.5. Model Assumptions, Limitations, and Future Perspectives

The proposed computational framework effectively demonstrates the utility of explicitly resolving the meso-scale anatomy (i.e., filament geometries and intra-/inter-layer cohesive interfaces) of 3D-printed concrete to reproduce slip-friction and topological toughening behaviors. However, the findings must be interpreted within the context of several core limitations.

First, the current framework relies on idealized geometric assumptions—perfect filament deposition, uniform cross-sections, and homogenously assigned cohesive properties. Consequently, in the defect sensitivity analysis (Section 5.3), structural flaws were represented solely by uniformly reducing the cohesive strength. This approach serves purely as a phenomenological proxy for homogenized interfacial damage. Real 3DCP interfaces inevitably exhibit discrete stochastic spatial variability, including micro-voids, filament waviness, nozzle offsets, and layer-height fluctuations. Explicitly simulating these stochastic geometric anomalies requires complex probabilistic mesh generation [39,40,41], which falls beyond the scope of this idealized topological study but represents a critical next step for comprehensively evaluating structural reliability. Given these idealizations, the computationally predicted 16.3-fold toughness enhancement for the 15° Bouligand architecture should be strictly interpreted as an upper-bound performance limit. Additionally, the model currently omits time-dependent phenomena (creep, shrinkage, and hydration evolution) and utilizes a single quasi-static loading rate.

Second, regarding experimental validation, physical testing was deliberately restricted to the 0° baseline and the 15° configuration with a limited sample size (*n* = 3) to establish a fundamental transition from brittle to pseudo-ductile behavior. The claimed optimum topological window (15–30°) and the associated stress redistribution mechanisms are derived entirely from numerical parametric sweeps. Extending physical validation to high pitch angles (e.g., 60°, 90°) remains experimentally challenging; continuous extrusion with severe orientational misalignments introduces compounding rheological defects (e.g., overhang gravitational collapse) [48] that can obscure the intrinsic topological toughening effect. Validating these high-angle configurations with larger sample sizes and rigorous statistical power is required.

Finally, the mechanistic interpretations of internal crack kinematics—such as 3D helical twisting, branching, and step-wise out-of-plane deflection—are numerical phenomena extracted directly from the CZM/CDP framework. Direct in situ experimental observations of these internal trajectories within dense, highly brittle cementitious matrices remain a significant experimental bottleneck. To bridge this gap, future investigations must integrate advanced non-destructive spatial imaging techniques, such as in situ X-ray micro-computed tomography (*μ*-CT) during compression, alongside digital image correlation (DIC) for full-field surface strain mapping [49,50]. These tools will be essential to physically observe the predicted 3D crack kinematics and further calibrate the discrete cohesive assumptions.

Notwithstanding these limitations, the principal finding—that the meso-scale spatial arrangement of 3D-printed filaments can dramatically alter the macroscopic failure mode and energy absorption capacity—has significant implications for digital concrete construction. This geometry-driven strategy complements, rather than replaces, conventional material-level reinforcement.

## 6. Conclusions

This study established a computation-driven mechanistic investigation to evaluate the fracture mechanisms of 3D-printed cementitious metamaterials with Bouligand architectures under compressive loading. By integrating nano-clay-assisted rheological modulation with an explicitly filament-resolved meso-scale cohesive zone model (CZM), the structural toughening effects of spatial toolpath design were systematically quantified. The main conclusions are as follows:

(1) Rheology-Enabled Shape Retention: The incorporation of nano-clay facilitated rapid thixotropic structural recovery (<0.2 s). This transient sol-to-gel transition enabled the support-free printing and high-fidelity shape retention of complex helicoidal topologies, providing the necessary physical basis for the idealized geometries utilized in the numerical models.
(2) Brittle-to-Pseudo-Ductile Transition and Energy Dissipation: The explicitly discretized meso-scale models, anchored by physical compression tests, successfully captured the transition from brittle longitudinal splitting in the 0° control to pseudo-ductile progressive damage in the 15° Bouligand architectures. Quantitatively, the physical 15° configuration exhibited a statistically significant (*p* < 0.01) 13.7-fold increase in volumetric work of fracture (*W*_f_) with a modest ~12% reduction in ultimate compressive strength compared to the 0° baseline. This aligns with the 16.3-fold toughness enhancement and ~11% strength reduction predicted numerically, which strictly serves as an upper-bound performance estimate.
(3) Numerically Predicted Crack Kinematics and Topological Optimization: The computational framework revealed that the orientational mismatch in Bouligand architectures induces 3D helical crack twisting, step-wise branching, and out-of-plane deflection. Based on these numerically derived internal kinematics, a parametric sweep identified a theoretical optimal topological window at pitch angles of 15–30°, wherein the competing effects of interfacial crack deflection and structural load-bearing capacity are optimally balanced.
(4) Imperfection Sensitivity and Defect Tolerance: Numerical parametric studies suggest that the topological toughening effect exhibits a degree of insensitivity to uniform interfacial strength reduction. The step-wise mixed-mode crack propagation increases the overall fracture path tortuosity, effectively compensating for local bond degradation. Under the idealized assumptions of the FEA framework, even with a 30% reduction in interfacial bonding strength, the 15° architecture retained a work of fracture approximately 12.4 times higher than that of the pristine 0° control.
(5) Limitations and Future Perspectives: These findings are based on idealized geometric assumptions (perfect filament dimensions, uniform bonding) and are primarily validated under compressive loading with a limited sample size (*n* = 3). The computationally predicted toughening metrics and the 15–30° optimal window should be interpreted as theoretical estimates demonstrating the potential of topological design rather than guaranteed practical limits. Future research must prioritize: (i) rigorous experimental validation of the optimal pitch angles using larger sample sizes (*n* ≥ 5) across diverse loading conditions (e.g., flexure, direct tension, and impact); (ii) the application of in situ X-ray *μ*-CT imaging to physically verify the numerically predicted 3D crack kinematics; and (iii) the extension of the computational framework to incorporate statistically representative stochastic geometric defects (e.g., filament waviness and local porosity) to capture the true variability inherent to 3D concrete printing.

## Figures and Tables

**Table 1:** Comparative summary of prior studies on Bouligand-inspired and architected cementitious materials.

Study	Material/System	Pitch Angles Tested	Modeling Approach	Experimental Validation	Key Limitations
Moini et al. (2018) [6]	Cement paste with fiber reinforcement	0°, 15°, 30°, 45°, 60°, 90°	Experimental only; no FEA	3-point bending; *n* ≥ 3	No interfacial CZM; no systematic FE parametric study
Suksangpanya et al. (2018) [8]	Polymer-matrix composite	0°, 15°, 30°, 45°	Analytical + FE with cohesive surfaces	Mode-I fracture on 3D-printed polymer	Polymer matrix; not validated for cementitious systems
Yang et al. (2025) [9]	3D-printed concrete; saddle-stitching	0°, 90° (cross-ply); no helicoidal sweep	Experimental only; limited FEA	Compression + flexure; *n* = 3–5	No Bouligand pitch-angle sweep; no defect sensitivity
Fan et al. (2026) [7]	Review—various cementitious	Various (reviewed)	Review of existing models	Review of reported experiments	Review article; no new data
To et al. (2024) [12]	3D-printed concrete; interlayer bond	0° only (unidirectional)	FEA with CZM (macro-scale)	Compression + flexure; *n* = 3	No Bouligand architecture; no pitch-angle sweep
Wolfs et al. (2019) [13]	3D-printed concrete; interlayer adhesion	0° only (unidirectional)	Experimental only	Compression + splitting tensile	No FEA; no bio-inspired architecture
Marchment & Sanjayan (2020) [14]	3D-printed concrete; mesh reinforcement	0° only (unidirectional)	Experimental only	Flexural testing	Reinforcement- based; no topological design
Present study	Cementitious ink + nano-clay; Bouligand	0–90° (full sweep)	Meso-scale FEA: C3D8R+ COH3D8, CDP + B-K CZM	Compression (0°, 15°); CZM calibration	Limited exp. validation (0°, 15°); idealized geometry; *n* = 3

**Table 2:** Chemical compositions of the Ordinary Portland cement.

Component	CaO	SiO_2_	Al_2_O_3_	Fe_2_O_3_	SO_3_	MgO	K_2_O	L.O.I.
Content (wt%)	62.83	20.50	5.61	3.84	3.07	1.70	1.31	1.14

L.O.I.: Lost on ignition.

**Table 3:** Experimental Test Program. All tests conducted after 28 days of standard curing (>95% RH, 20 ± 2 °C).

Test Type	Standard	Specimen Dimensions	Configurations	Replicates	Loading Rate
Rheological characterization (3ITT, flow curve)	RILEM TC 266–MRP [30]	~5 mL sample	NM-modified ink	3	0.01–200 s^−1^ (sweep)
Zeta potential	ISO 13099–2 [31]	~1 mL diluted suspension	NM-modified ink	3	—
Uniaxial compression	ASTM C39/C39M–21 [32]	Ø50 × 100 mm (cores)	0°, 15°	3	0.5 mm/min (disp.)
Splitting tensile (CZM Mode–I calibration)	ASTM C496/C496M–17 [33]	Ø50 × 25 mm (cores)	2-layer printed	3	0.05 mm/min
Direct shear (CZM Mode–II calibration)	—	50 × 50 × 25 mm (sawn)	2-layer printed	3	0.02 mm/min

**Table 4:** Summary of material constitutive parameters and interfacial cohesive properties used in the FEA models (Interfacial properties were derived and calibrated from Refs. [11,22,23] along with preliminary internal testing).

Category	Parameter	Symbol	Value	Unit
Bulk Matrix (CDP)	Density	*ρ*	2350	kg/m^3^
	Young’s modulus	*E*	24.5	GPa
	Poisson’s ratio	*ν*	0.2	--
	Compressive yield strength	*f*_c0_	33.0	MPa
	Dilation angle	*ψ*	36	deg
	Eccentricity	*e*	0.1	--
	Biaxial/uniaxial strength ratio	*f*_b0_ */f*_c0_	1.16	--
	Deviatoric cross-section shape factor	*K*_c_	0.667	--
	Normal peak strength	*τ*_n_	2.8	MPa
	Shear peak strengths	*τ*_s_, *τ*_t_	4.2	MPa
	Mode I fracture energy	*G*_IC_	0.06	N/mm
	Mode II/III fracture energy	*G*_IIC_, *G*_IIIC_	0.32	N/mm
	Power-law constant (B-K)	*η*	1.6	--

**Table 5:** Comparison of experimental and FEA results (mean ± SD, *n* = 3). The systematic FEA over-prediction (+9–11% for *σ*_max_; +9–29% for *W*_f_) is attributable to the idealized model geometry (no micro-pores, uniform interfacial bonding, perfect filament dimensions).

Configuration	Exp. *σ*_max_ (MPa)	FEA *σ*_max_ (MPa)	Δ*σ* (%)	Exp. *W*_f_ (MJ/m^3^)	FEA *W*_f_ (MJ/m^3^)	Δ*W*_f_ (%)	Toughness Ratio (vs. 0° Exp.)
0° (unidirectional)	30.2 ± 1.5 (CoV: 5.0%)	33.0	+9.3%	0.045 ± 0.008 (CoV: 17.8%)	0.049	8.9%	1.0× (baseline)
15° (Bouligand)	26.5 ± 2.1 (CoV: 7.9%)	29.5	+11.3%	0.620 ± 0.042 (CoV: 6.8%)	0.80	29.0%	13.7× (experimental) 16.3× (numerical)

**Table 6:** Parametric sweep results—effect of pitch angle on strength and energy absorption.

Pitch Angle *γ* (°)	FEA *σ*_max_ (MPa)	FEA *W*_f_ (MJ/m^3^)	Toughness Ratio (vs. 0° FEA)	Experimental Validation
0°	33.0	0.049	1.0× (baseline)	*n* = 3 (*σ*_max_ = 30.2 ± 1.5 MPa, *W*_f_ = 0.045 ± 0.008 MJ/m^3^)
15°	29.5	0.80	16.3×	*n* = 3 (*σ*_max_ = 26.5 ± 2.1 MPa, *W*_f_ = 0.62 ± 0.042 MJ/m^3^)
30°	27.8	0.64	13.1×	Numerical prediction only
45°	24.2	0.38	7.8×	Numerical prediction only
60°	22.1	0.26	5.3×	Numerical prediction only
90°	20.5	0.15	3.1×	Numerical prediction only

**Table 7:** Imperfection sensitivity analysis—effect of interfacial bond-strength reduction on the 15° Bouligand architecture (numerical predictions only).

Bond Strength Reduction (%)	*σ*_max_ (MPa)	*W*_f_ (MJ/m^3^)	Toughness Retention Ratio (vs. Control)
0% (Baseline)	29.5	0.80	16.3×
10%	28.0	0.74	15.1×
20%	26.5	0.68	13.9×
30%	25.0	0.61	12.4×

## Data Availability

The original contributions presented in this study are included in the article/Supplementary Material. Further inquiries can be directed to the corresponding author.

## Supplementary Materials

The following supporting information can be downloaded at: https://www.mdpi.com/article/10.3390/ma19132892/s1, Figure S1: Global energy balance verification for the quasi-static explicit analysis. The evolution of internal energy (ALLIE) and kinetic energy (ALLKE) is plotted against normalized simulation time. Throughout the deformation process, the kinetic energy remains bounded below the 5% ALLIE threshold, with a maximum ratio of approximately 0.4%. This energetic metric confirms that the macroscopic fracture behavior is driven by strain energy dissipation rather than artificial inertial effects.
