# Supplementary material for: A Meso-Scale Computational Framework for Predicting Fracture Mechanisms in 3D-Printed Bouligand Cementitious Metamaterials
Source: Materials (Basel). 2026 Jul 6;19(13):2892. doi: 10.3390/ma19132892 (PMC13362796; doi:10.3390/ma19132892)
Supplement: Supplementary file 1 [file materials-19-02892-s001.zip › materials-4365463-supplementary.pdf]

Supplementary Information for "A Meso-scale Computational Framework for Predicting Fracture Mechanisms in 3D-Printed Bouligand Cementitious Metamaterials"

Xuelian Yuan *, Yaqing Jiang and Huiting Xiong

School of Civil Engineering, Wanjiang University of Technology, Ma'anshan 243031, China
* Correspondence: xue_lian_yuan@163.com

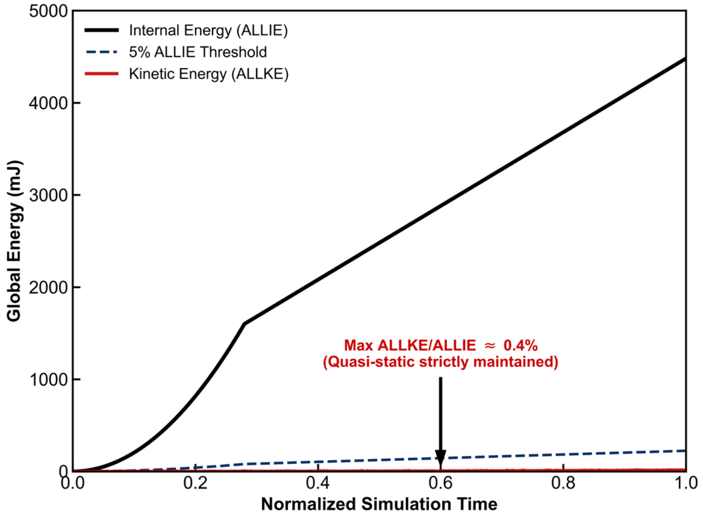

Figure S1. Global energy balance verification for the quasi-static explicit analysis. The evolution of internal energy (ALLIE) and kinetic energy (ALLKE) is plotted against normalized simulation time. Throughout the deformation process, the kinetic energy remains bounded below the 5% ALLIE threshold, with a maximum ratio of approximately 0.4%. This energetic metric confirms that the macroscopic fracture behavior is driven by strain energy dissipation rather than artificial inertial effects.
